# Enhancing Chinese EFL Teachers’ Work Engagement: The Role of Self and Collective Efficacy

**DOI:** 10.3389/fpsyg.2022.941943

**Published:** 2022-07-11

**Authors:** Liping Ge

**Affiliations:** School of Foreign Languages, Henan University of Technology, Zhengzhou, China

**Keywords:** collective efficacy, self-efficacy, work engagement, EFL teachers, EFL (English as a foreign language)

## Abstract

Teachers’ work engagement is considered an essential component in instruction. Accordingly, the emphasis should be over physical and mental predictors of this construct. In this line, this study investigates the relationship between Chinese English as a Foreign Language (EFL) teachers’ individual self-efficacy, collective efficacy, and work engagement. To this end, 300 Chinese instructors (males = 96, females = 204) from different colleges and universities participated in this study. The questionnaires were distributed among teachers with different educational levels and experiences. Linear multiple regression was used as a measure for data analysis. The findings showed the significant correlations between teachers’ work engagement, self-efficacy, and collective efficacy. Comparing the predictability power, teachers’ self-efficacy (*B* = 0.57) proved to have a higher index compared to their index of collective efficacy competence (*B* = 0.22). This study concluded that self-efficacious teachers and teachers who believe in collective efficacy are more engaged in the EFL contexts. Moreover, the study has some pedagogical implications and suggestions for different teacher educators, administrators, and advisors.

## Introduction

It should be underlined that instructors have their individual personality traits, principles, reasoning, and reflection that considerably influence their teaching activities in educational contexts (Kim et al., 2019). Numerous studies have proved that instructors’ insights, perceptions, and affections significantly correlate with learners’ academic success (Klassen and Tze, 2014). Consequently, many studies on teachers’ individual efficacy, job involvement, teacher burnout, teacher boredom have drawn the attention of multiple investigators.

The concept of teachers’ work engagement, in educational contexts, is significant but disregarded in the conventionalized EFL classroom contexts (Zhao et al., 2021). In order to succeed and survive in a continuously changing and competitive environment, schools need motivated proactive and initiative teachers who collaborate. In fact, schools that try to engage teachers who have an affective bond with their professional activities surpass others. The concept of work engagement was first coined by Kahn in 1990, who defined it as the “harnessing of organization members’ selves to their work roles. In engagement, people employ and express themselves physically, cognitively, emotionally, and mentally during role performances” (p. 694). In fact, it is the extent to which an individual is attentive and absorbed in the performance of his or her work. According to Kahn (1990), when people are engaged, they are not only physically involved in their work, but also are cognitively alert and emotionally connected to others at the moment of engagement. Educators with higher levels of engagement in their job are inclined to be active, devoted, and fascinated by the educational contexts (Seligman, 2011). Work engagement, as a positive psychological construct, has drawn the attention of main investigators in recent years (Burić and Macuka, 2018).

According to Schaufeli et al. (2002), teachers’ work engagement refers to “a positive, fulfilling, work-related state of mind that is characterized by vigor, dedication, and absorption” (p. 202). They argued that dedicated and absorbed instructors can provide inspiring educational contexts in which learners tend to engage in the learning process. Teachers’ work engagement can predict their teaching effectiveness, activities, problem-solving, and job satisfaction (Minghui et al., 2018).

Bandura (1986) defined self-efficacy as “people’s judgments of their capabilities to organize and execute courses of action required to attain designated types of performances” (p. 391). He stated that self-efficacy pinpoints individuals’ self-reliance on their competence to cope with challenging tasks, and to put into practice the prerequisite strategies to be successful in impending situations. In the instructional environment, an individual’s self-efficacy is generally measured by teacher self-efficacy (Corry and Stella, 2018). According to Miller et al. (2017), teachers’ self-efficacy is regarded as the self-confidence among teachers in changing learners’ learning capability. To boost teacher self-efficacy, Bandura (1997) indicated four principal methods, including mastery experiences, vicarious experiences, social persuasion, and physiological and emotional states.

Bandura (1997) also highlights the prominence of strengthening the principles that individuals can prosper in their working contexts through cooperation to cope with upcoming challenges. He introduced the concept of teachers’ collective efficacy. Tschannen-Moran and Barr (2004) defined teachers’ collective efficacy as “educators’ shared beliefs that through their combined efforts they can positively influence student outcomes, including those who are disengaged, unmotivated, and/or disadvantaged” (p. 190) Guidetti et al. (2018) argued that teachers’ collective and self-efficacy is on the premise that school, in total, can carry out and establish courses of actions influencing learners and their achievements. They stated that collective efficacy principles highlight the group’s functioning competencies.

These variables show that educators’ features are worth investigating to enhance their teaching activities. Conventionally, EFL educators’ traits have been estimated based on their foreign language knowledge, qualifications, and experience. Nevertheless, studies have also recognized the significance of educators’ approaches, viewpoints, and principles about their instruction to expedite learners’ academic achievement (Ekstam et al., 2017). So, examining factors like work engagement and the effect of self-efficacy on it, can shape teachers’ concepts. Moreover, there are limited studies on research on these subjects in China. Investigators have proved that work engagement can strongly predict learners’ performance and low burnout among teachers (Burić and Macuka, 2018). A challenging problem that arises in this domain is that investigators are still studying if teachers’ individual self-efficacy is sufficient to predict work engagement (Klassen and Tze, 2014). There are very few investigations (e.g., Li et al., 2017; Chan et al., 2020; Bao et al., 2021; Han and Wang, 2021) about teacher self-efficacy and teacher work engagement in the Chinese EFL context. The outcomes of not investigating this problem lead to wasted opportunities for learner academic development. This study aims at investigating the relationship between, self-efficacy, teacher-efficacy and teacher engagement in Chinese context.

This study seeks to answer the following questions:

Q1: Is there any significant relationship between Chinese EFL teachers’ individual efficacy, collective efficacy, and work engagement?

Q2: To what extent, do Chinese EFL teachers’ individual self-efficacy and collective efficacy predict work engagement?

## Review of Literature

### The Related Studies on Teacher Self-Efficacy

Zee et al. (2018) found that teacher self-efficacy is considered as one of the most important facets of instruction that strongly predicts teaching effectiveness and learners’ academic achievement. Klassen and Tze (2014) asserted that self-efficacious teachers outperform in organizing and preparing educational contexts. They are more dedicated to their job to know the requirement of learners. Moreover, they embrace innovations, keep on their profession, and deal with troublesome (Klassen and Tze, 2014). Investigations have indicated that self-efficacious teachers generate highly energetic educational contexts with superior lesson planning, profound teaching, and operational classroom control (Chao et al., 2017).

Studies have shown that self-efficacy can be regarded as a convincing basis for reducing negative emotions like foreign language anxiety, and arousing positive psychology constructs, such as well-being and resilience. Hülya et al. (2018), in their study, revealed that teaching apprehension was an important negative issue influencing self-efficacy. They mentioned that instrumental support and care from teacher educators and school principals could compensate for typical causes for attrition, including substantial assignments, student behavioral matters, and general anxiety. Troesch and Bauer (2017) also found out that teacher self-efficacy significantly reduces job stress. Ghasemzadeh et al. (2019) found out that teacher self-efficacy, compared to teacher self-reflection, significantly predicts teacher burnout. They argued that teachers who feel less competent in classroom control can feel higher job pressure, which may increase emotional distress and depersonalization. They mentioned that instructors, who suffer depersonalization, no longer show their feelings toward learners. They also justified their study by expressing that the fundamental constituents of teacher self-efficacy are said to predict teacher burnout. They also mentioned that instructors, with high levels of self-efficacy, can innovatively develop and apply various methodologies and teaching techniques, and they can involve learners in order to overcome depersonalization. In line with Ghasemzadeh et al. (2019); Fathi et al. (2021a) also revealed that EFL teachers’ self-efficacy, emotional control, and reflection result in the reduction of burnout experiencing in educational contexts and ineffective teaching. They suggested that EFL teachers should participate in preparation agendas to foster their self-efficacy, reduce stressful situations, and to alleviate the likelihood of teacher burnout.

Concerning positive emotions, Fathi et al. (2020) found a significant relationship between self-efficacy and psychological well-being. They mentioned that job satisfaction and work obligation, along with less anxiety or burnout, are the results of self-efficacy and well-being. Based on Bandura’s (1986) theory, they justified that individuals’ opinions affect both their performance in tasks and thinking patterns and feelings as the critical constructs of psychological well-being. Huang et al. (2019), in their study, demonstrated that teacher collective efficacy significantly influences teacher well-being. Concerning resilience as a positive emotion, the findings of Razmjoo and Ayoobiyan (2019) revealed that numerous fundamental constituents of self-efficacy were significantly correlated with teacher resilience. Regarding grit, Jiang et al. (2021) found out that self-efficacy can mediate the relationship between grit and cognitive learning strategy in Chinese EFL contexts. In another study in Chinese and Iranian contexts, Yang et al. (2022) tested a hypothesized model of the interrelationship among variables using Mplus. According to this model, L2 grit was significantly influenced by learners’ academic buoyancy and self-efficacy. Their findings implied that efficacious and buoyant students are more likely to be gritty, and to cultivate L2 grit, the teachers should encourage efficacious and buoyant EFL learners by attending to students’ emotional and motivational drives instead of merely cognitive ones.

Earlier studies have shown that teacher self-efficacy can significantly predict teacher motivation and educating behaviors. Individuals with high levels of self-efficacy are highly motivated, and have individual achievements (Engin, 2020). Demirtas (2018) argued that teachers with high levels of self-efficacy strongly rely on their capability to accomplish in challenging situations, make the demanding decision for their success, and cope with disappointing experiences. Heuven et al. (2006) stated that efficacious instructors are motivated to employ resources in educational contexts to manage arduous activities appropriately. Work engagement, as a component of positive psychology and motivational construct, has been regarded as one of the important issues in the field of instruction, and its relationship with teacher self-efficacy should be considered in more detail.

### The Related Studies on Teacher Work Engagement

Teacher engagement is regarded as a motivational concept signifying instructors’ voluntary allocation of physical, cognitive, and emotional resources across teaching-related activities (Klassen et al., 2013). This definition is based on multidimensional conceptualization of teacher engagement proposed by Klassen et al. (2013), comprising cognitive-physical, emotional, and social dimensions. Cognitive-physical engagement is the extent to which teachers attend to and invest effort in work tasks. Emotional engagement refers to teachers’ positive emotional responses to their work. Finally, social engagement, comprising both student and colleague domains, refers to teachers’ perceptions of their connection to, and concern for, students and colleagues, respectively. The conceptualization of cognitive-physical and emotional engagement in Klassen et al. (2013) can also be traced to Schaufeli et al.’s (2002) perspective who defined work engagement as a “positive, fulfilling, work-related state of mind that is characterized by vigor, dedication, and absorption” (p. 74). A novel aspect of Klassen et al.’s (2013) conceptualization, relative to previous models of work engagement, is the addition of social dimensions of engagement. Klassen et al. (2013) justified this conceptual addition by arguing that existing models of work engagement do not sufficiently account for teachers’ investment of energy in establishing connections with students and colleagues.

Few studies (e.g., Nayyar et al., 2013; Li et al., 2019; Topchyan and Woehler, 2020, etc.) have investigated the relationship between teachers’ work engagement and demographical variables, and personality traits. Topchyan and Woehler (2020) investigated the gender effect and teaching experience on teacher engagement and job satisfaction in educational contexts. Their study revealed that female instructors were more engaged in instructional contexts. Moreover, they found that teaching experience is significantly correlated with job satisfaction and work engagement. Concerning teachers’ personality traits, Nayyar et al. (2013) highlighted the role of “extraversion,” “agreeableness,” “conscientiousness,” and “openness to experience” in influencing teachers’ work engagement.

The relation between work engagement and negative and positive emotional constructs has been discussed in a few studies. Regarding negative emotional construct, Sonnentag et al. (2008) found a significant negative correlation between work engagement and emotional exhaustion. Faskhodi and Siyyari (2018), in their study, revealed that teacher burnout and exhaustion, as negative emotional factors, significantly predict teachers’ work disengagement. Concerning positive emotional constructs, Zhang and Yang (2021), in their study, indicated that teachers’ job engagement is significantly correlated with learners’ academic engagement in EFL classroom environments. They argued that engaged learners are inclined to provide opportunities for instructors to be dedicated and absorbed in the instruction. Greenier et al. (2021) showed that teachers’ well-being and emotional regulation strategies significantly correlate with teacher engagement. They argued emotional regulation strategies used by teachers are adequate for their involvement in doing educational tasks. Zeng et al. (2019), in their study in a Chinese context, demonstrated that teachers’ growth mindset, well-being, and resilience strongly predict job engagement. They also found out that well-being and grit mediate the correlation between work engagement and growth mindset. Lee (2019), in his study, found out that teachers’ positive emotions such as self-esteem and positive attitude are significantly correlated with job engagement. This study tries to investigate the relationship between other two positive constructs like self-efficacy, collective efficacy and work engagement.

### The Relationship Between Teacher Self-Efficacy, Collective Efficacy, and Work Engagement

Some studies have been done on teacher self-efficacy and work engagement. Granziera and Perera (2019) did not find a direct relationship between teacher self-efficacy and work engagement. They argued that since the association between teacher self-efficacy and job satisfaction depends on role-based tasks, teacher engagement can mediate the relationship between job satisfaction and self-efficacy. They used the social cognitive career theory model, proposed by Lent and Brown (2006), to justify their findings. They argued that instructors’ self-efficacy is associated with teachers’ involvement in tasks, and teacher engagement is an indication for engaging in goal-iriented activities. In line with Granziera and Perera (2019); Wang (2021) also found that the attribute of self-efficacy can significantly influence the relationship between teacher engagement and learners’ academic success. He argued that self-efficacious instructors seem to regard instructional difficulties as a manageable issue and are inclined to employ innovative methodologies to contribute their learners to accomplishing their tasks.

Kong (2021), on the other hand, found a direct correlation between teacher engagement and self-efficacy. His study showed that Chinese EFL educators’ job involvement is significantly influenced by their well-being and efficacy. He concluded that self-efficacious EFL teachers with high levels of well-being are inclined to show higher levels of involvement in instructional contexts. Likewise, Han and Wang (2021) investigated the relationship between Chinese teacher self-efficacy and work involvement in educational contexts. Their findings indicated that the construct of teacher self-efficacy is entangled with work involvement. Sokmen and Kilic (2019), in their study, found out that teacher self-efficacy significantly predicted job involvement, teacher satisfaction, and self-sufficiency, whereas it was negatively correlated with teacher burnout. They used Bandura’s (1997) social learning theory to explicate their results in that self-efficacy is considered as a feature that diminishes teacher apprehension and provokes job engagement. Using job demands-resources as a model of studying, Huang et al. (2016) showed the significant effect of self-efficacy and positive attitude on teacher engagement. Li et al. (2019) investigated the effect of continuing professional development and years of instructional experience on teachers’ work engagement and self-efficacy. They found out that job involvement is significantly correlated with teacher self-efficacy. Their study also showed the strong predictability power of continuing professional development among novice instructors in work engagement and self-efficacy. Furthermore, their study showed that novice teachers’ involvement in innovative and engaging tasks is significantly correlated with their efficacy.

Task type is also effective in the extent of engagement. Grigg et al. (2018) found out that engagement in particular tasks provides the opportunity for the instructors to develop their teaching capability and self-efficacy. The study of Lipscomb et al. (2021) revealed that teachers’ self-efficacy and education are significantly correlated with teacher work engagement. They stated that self-efficacious teachers who have confidence in their ability to teach effectively to the elementary learners are eagerly involved in their job. They maintained that the performance of these teachers is described by devotion, vigor, and absorption. They also asserted that teacher self-efficacy is considered as a significant element of individual resource that helps them to keep on performing with eagerness and commitment to educational contexts. To determine the reciprocity of the relationship between teacher engagement and self-efficacy, Burić and Macuka (2018) found out that involved instructors regarded themselves as more efficacious. Using the 4-point Likert scale and Utrecht Work Engagement Scale, they found out that vigor, dedication, and absorption are significantly and mutually correlated with teacher self-efficacy. They suggested that school principals ought to utilize techniques that expand instructors’ self-efficacy and endorse positive emotions which influence work involvement.

In a study on the relationship between teacher collective efficacy and work engagement, Skaalvik and Skaalvik (2019) found an indirect relationship between these two constructs. However, teacher self-efficacy mediated in this correlation. Huang et al. (2019) also argued that teachers’ trust in associates, as an indicator of collective self-efficacy, significantly affected their psychological well-being and engagement by endorsing self-efficacy. Fathi et al. (2021b) compared the effects of self-efficacy and collective efficacy on teacher work engagement. They found that the predictability power of teacher self-efficacy, compared to collective efficacy, in work engagement is higher. They argued that self-efficacious instructors are more involved in their job by determining appropriate educational objectives. They also maintained that awareness of collective efficacy among teachers enhances self-efficacy, which fosters instructors’ robustness, devotion, and fascination in their instruction. They asserted that instructors who believe in collective efficacy are more enthusiastic and emotionally buoyant during their work and dedicate themselves to their instruction in coping with challenging situations. In general, more studies are still required to elucidate the correlation between teachers’ self-efficacy, collective efficacy, and work engagement.

## Methodology

### Participants

The sample (as shown in Table 1) comprised of 300 teachers from different colleges and universities in China, including both genders (males = 96, females = 204) with their age ranging from 31 to 60. Their average ages was 47.3. They are with different educational qualifications and various years of teaching experiences with 1 to 5 years (18), 6 to 10 years (25), 11 to 15(37), 16-20(92), 21-25(79) and 26 + (49) accounting for 6, 8.33, 12.33, 30.67, 26.33, and 16.33%, respectively. Most of them 67.3% had obtained the degree of the Master of Arts. They were collected from different provinces of China, with the majority in Henan Province (75.67%) and the rest are from 8 other provinces (23.6%) and 2 municipalities directly (Beijing, Shanghai/0.73) under the central government. Consent had been given to all participants before they participated in this study. All responses were based on their willingness.

**TABLE 1:** Demographic information.

Years of experience	Number of teachers
1-5 years	18
6-10 years	25
11-15 years	37
16-20 years	92
21-25 years	79
More than 26 years	49

### Data Collection Procedures

The data were collected over the week, from February 5, 2022, to February 12. Altogether, researchers obtained 300 valid questionnaires. To ensure the reliability and validity of the study, the questionnaire was carefully made, translated into Chinese and then examined for any potential mistake before being launched into Wenjuanxing, a widely used program in China to collect the data. To generalize the results of this study, the questionnaires were distributed into different cities and were completed by teachers with different educational levels and experiences. All participants were informed of how to fill properly the questionnaires with their own electronic devices, and what they can do to prevent any exception in the process of study. They had the freedom and right of withdraw from the study if they sensed any discomfort in the study. Then, collected data was double-checked before being sent into SPSS for further exploration, which finally laid the foundation for the probe into the research questions.

## Results

To decide on the data analysis, preliminary measurements should be done. The first step is to measure the reliability of the three questionnaires used in this study.

As shown in Table 2, the process of calculation was repeated three times to measure the reliability indices of all three questionnaires, and the outputs of Cronbach’s alpha revealed that the work engagement questionnaire (*r* = 0.95), self-efficacy questionnaire (*r* = 0.97), and collective efficacy questionnaire (*r* = 0.92) had satisfactory reliability indices.

**TABLE 2:** Reliability of the questionnaires.

Questionnaire	Cronbach’s alpha	No. of Items
WE	0.95	17
SE	0.97	24
CE	0.92	7

One of the ways the researcher used for making decisions on using parametric or non-parametric analysis in a quantitative study, is to measure the normality of the data. Table 3 shows the Kolmogorov-Smirnov index which shows that the distribution of data is normal for WE (0.05), and SE (0.07), but not for CE (sig = 0.000). The assumption for having a standard set of data is to have a non-significant index of K-S, but the output revealed that the data normality rule is violated for collective efficacy, and a non-parametric analysis should be conducted to calculate the possible relationships among the variables.

**TABLE 3:** Test of normality for WE, SE, and CE.

	Kolmogorov-Smirnov*^a^*			Shapiro-Wilk		
	Statistic	Df	Sig.	Statistic	df	Sig.
WE	0.051	300	0.058	0.972	300	0.000
SE	0.049	300	0.079	0.972	300	0.000
CE	0.102	300	0.000	0.972	300	0.000

*a, Lilliefors Significance Correction.*

### The First Research Question

The first research question was posed to measure the possible relationship between Chinese EFL teachers’ work engagement, self-efficacy, and collective efficacy. Since it was revealed in the Table 3 that the data are not normal, a non-parametric correlation index was used.

Based on the correlational rules, the greater the correlation factor, the stronger the possibility of a significant relationship. As shown in Table 4, the relationship between teachers’ work engagement and self-efficacy is direct and significant (*r* = 0.732, *p* = 0.000). Similarly, the relationship between teachers’ work engagement and collective efficacy is direct and significant (*r* = 0.602, *p* = 0.000). It can be concluded that the higher the level of teachers’ work engagement, the higher their level of self and collective efficacy.

**TABLE 4:** Correlation among for WE, SE, and CE.

			WE	SE	CE
Spearman’s rho	WE	Correlation Coefficient	1.000	0.732**	0.602**
		Sig. (2-tailed)	.	0.000	0.000
		N	300	300	300
	SE	Correlation Coefficient	0.732**	1.000	0.705**
		Sig. (2-tailed)	0.000	.	0.000
		N	300	300	300
	CE	Correlation Coefficient	0.602**	0.705**	1.000
		Sig. (2-tailed)	0.000	0.000	.
		N	300	300	300

**, Correlation is significant at the 0.01 level (2-tailed).

### The Second Research Question

The second research question concerns the extent to which Chinese EFL teachers’ self and collective efficacy can predict work engagement. This measurement was done by running a multiple regression analysis. The following tables were the output of linear multiple regression analysis, including, model summary, ANOVA, and coefficient.

As seen in Table 5, based on the number of items and the value of Likert type, the mean scores, and standard deviation indices were calculated.

**TABLE 5:** Descriptive statistics for WE, SE, and CE.

Variables	M	SD	N
WE	90.25	17.004	300
SE	112.28	16.08	300
CE	26.19	5.07	300

Table 6 provides a model summary for Chinese EFL teachers’ WE, SE, and CE. It was shown that the model, which contains the scores of teachers’ SE and CE, can explain the amount of variance in the dependent variable (teachers’ work engagement). This model can explain 55.2% of the variances in the teachers’ work engagement.

**TABLE 6:** Model summary for WE, SE, and CE.

Model	R	R Square	Adjusted R Square	Std. Error of the Estimate
1	0.743	0.552	0.54	11.42

Table 7 labeled ANOVA test of the hypothesis that multiple R in the population equals zero (0). The model reached statistical significance (*F* = (2, 297) = 182.91, Sig = 0.000, this really means *p* < 0.05).

**TABLE 7:** ANOVA for WE, SE, and CE.

Model		Sum of Squares	df	Mean Square	F	Sig.
1	Regression	47718.70	2	23859.35	182.91	0.000
	Residual	38741.54	297	130.44		
	Total	86460.25	299			

To measure whether the independent variables (teachers’ self and collective efficacy) can predict the dependent variable (teachers’ work engagement), the sig. column was studied. As shown in Table 8, both independent variables are significant predictors. Comparing the predictability power, teachers’ self-efficacy (*B* = 0.57) proved to have a higher index compared to their index of collective efficacy competence (*B* = 0.22).

**TABLE 8:** Coefficients for WE, SE, and CE.

Model		Unstandardized Coefficients		Standardized Coefficients		Sig.
		B	Std. Error	Beta	T	
1	(Constant)	2.94	4.66		0.63	0.529
	SE	0.603	0.05	0.57	10.57	0.000
	CE	0.747	0.18	0.22	4.13	0.000

## Discussion and Conclusion

This study made an attempt to examine the prediction of Chinese EFL instructors’ self-efficacy and their collective efficacy for work engagement. It is revealed that teachers’ self-efficacy and collective efficacy are significantly correlated with work engagement. Moreover, the predictability power of teachers’ self-efficacy was higher than collective efficacy. Our findings hint that self-efficacious instructors tend to be more engaged in their instruction. Our findings are in accordance with findings reported by Huang et al. (2019), who showed that teachers’ belief in collective efficacy can significantly affect their involvement through improving individual self-efficacy. Moreover, the findings of this study are in line with Skaalvik and Skaalvik (2019) who did not find a straightforward relationship between these two constructs. However, in line with the ideas of Fathi et al. (2021a), it can be concluded that some factors, such as having self-confidence in setting up reasonable goals, resilience, grit, and striving for disentangling the issues, can be the reasons for the effectiveness of self-efficacy on robustness, devotion, and fascination in instruction. Teachers, having collective efficacy conceptions, tend to experience a feeling of efficiency, interest, motivation along self-importance when they involve in their job. A trait such as collective efficacy may encourage instructors to concentrate on their instruction.

Concerning the relationship between teacher individual efficacy and collect efficacy, this study confirmed the results of Han and Wang (2021), who proved the correlation between Chinese instructors’ individual self-efficacy and work engagement. The findings of this study tie well with the results of Burić and Macuka (2018), who indicated that teachers with high self-efficacy have sufficient knowledge of their abilities in the field of teaching and their job, and are with increased self-confidence when facing changes in job. Therefore, self-efficacious teachers find a positive mentality toward these changes, and this positive mindset reduces their resistance to change and boosts engagement. Generally, our results are in line with Skaalvik and Skaalvik (2019), who believed that “in the actual teaching situation, the teachers are primarily alone and must trust their own skills and abilities” (p. 1406). A similar pattern of results was obtained by Lipscomb et al. (2021) in that self-efficacious educators with positive attitudes toward their capabilities in employing operational methodologies, involving learners have a great tendency to involve in the instruction. This study is also consistent with Kong (2021), who mentioned that teachers with higher levels of self-efficacy can prevail over the teaching difficulties, put more effort into instruction, and have higher levels of grittiness in facing demanding issues. This research proves Granziera and Perera (2019), who found an inter-relatedness construct between job satisfaction, teacher individual efficacy, and job engagement. Moreover, other findings were broadly in line with Sokmen and Kilic (2019) who mentioned that teachers’ individual self-efficacy may foster obligation and accountability, which increases work involvement.

This study includes some pedagogical implications for teacher educators, educational policy-makers, and advisors. To improve individual and collective self-efficacy and work engagement, teacher educators and mentors can provide a situation in which teachers can observe the instruction of their peers. Teacher educators can also emphasize instructors to overemphasize on the constant academic development and critical thinking to enhance their instructional method. Instructors should be directed to be well-informed about instructive issues and take advantage of improved learning opportunities. It is also suggested that teacher educators highlight interactive tools, like mobile applications, which encourage teachers and learners to interact and scaffold that increase efficacy. They should develop confidence and competence among in-service teachers to entice learners’ interests and engage them in the learning process.

Educational policy-makers should hire experienced teachers, as the instructive experience can be an important issue for increasing efficacy among teachers. They can increase teacher self-efficacy by holding academic workshops that offer teachers some authentic activities. They can ask teachers to do their best within varied educational contexts. They must build up teaching effectiveness through providing contexts for observations of other teachers’ activities and mastery experiences to amplify general self-efficacy in particular ranges of the instruction. They should also provide critical thinking, creativeness, and motivation into the education in classrooms, which encourages work engagement. Consequently, the preparation of the contexts, and the vicarious observation may result in decreasing instructional challenges and enhancing instructors’ teaching performances. The importance of efficacy and engagement can motivate advisors to expand their horizons to identify teachers’ sources of efficacy and engagement to remove their barriers.

This study has some limitations. Most of the participants of this study are from one province and few from other cities. This can cause a generalization issue. Next, the number of participants in studies using this quantitative approach is often limited. A small number of teachers participated in this research. Beliefs and cognitions held by this sample of participants may not inevitably depict the cognitions of a larger population.

Future studies should aim to replicate results in larger contexts. In future work, investigating teachers’ individual self-efficacy and collective efficacy and its role in work engagement in technology-supported contexts, numerous cultural backgrounds, and among teachers with different educational experiences can be important for future studies. Some investigations need to be done on the effect of teachers’ individual and collective efficacy on learner motivation in traditional and virtual contexts. Furthermore, the relationship between teacher proficiency level of foreign language, and its effect on their work engagement and efficacy should be considered in future study. Moreover, case and phenomenological investigations, which provide us the reasons behind teachers’ individual and collective efficacy are required to be done. Some studies should be done on the relationship between positive psychological constructs such as enjoyment, grit, positive affectivity, resilience, and collective efficacy. In addition, future research should examine the roles of negative factors such as anger, frustration in self-efficacy, and work engagement. Some investigations should also be done on the effect of cross-cultural perspectives on teacher self-efficacy in expatriate instructors in Chinese educational contexts.

## Data Availability Statement

The original contributions presented in this study are included in the article/supplementary material, further inquiries can be directed to the corresponding author/s.

## Ethics Statement

The studies involving human participants were reviewed and approved by Henan University of Technology Academic Ethics Committee. The participants provided their written informed consent to participate in this study.

## Author Contributions

The author confirms being the sole contributor of this work and has approved it for publication.

## Conflict of Interest

The author declares that the research was conducted in the absence of any commercial or financial relationships that could be construed as a potential conflict of interest.

## Publisher’s Note

All claims expressed in this article are solely those of the authors and do not necessarily represent those of their affiliated organizations, or those of the publisher, the editors and the reviewers. Any product that may be evaluated in this article, or claim that may be made by its manufacturer, is not guaranteed or endorsed by the publisher.
